# Redox regulation of UPR signalling and mitochondrial ER contact sites

**DOI:** 10.1007/s00018-024-05286-0

**Published:** 2024-06-07

**Authors:** Jose C. Casas-Martinez, Afshin Samali, Brian McDonagh

**Affiliations:** 1 Discipline of Physiology, School of Medicine, University of Galway, Galway, Ireland; 2 Apoptosis Research Centre, University of Galway, Galway, Ireland; 3 School of Biological and Chemical Sciences, University of Galway, Galway, Ireland

**Keywords:** Hormesis, Redox signalling, Mitochondrial dynamics, Contact-sites, Skeletal muscle, *C. elegans*

## Abstract

Mitochondria and the endoplasmic reticulum (ER) have a synergistic relationship and are key regulatory hubs in maintaining cell homeostasis. Communication between these organelles is mediated by mitochondria ER contact sites (MERCS), allowing the exchange of material and information, modulating calcium homeostasis, redox signalling, lipid transfer and the regulation of mitochondrial dynamics. MERCS are dynamic structures that allow cells to respond to changes in the intracellular environment under normal homeostatic conditions, while their assembly/disassembly are affected by pathophysiological conditions such as ageing and disease. Disruption of protein folding in the ER lumen can activate the Unfolded Protein Response (UPR), promoting the remodelling of ER membranes and MERCS formation. The UPR stress receptor kinases PERK and IRE1, are located at or close to MERCS. UPR signalling can be adaptive or maladaptive, depending on whether the disruption in protein folding or ER stress is transient or sustained. Adaptive UPR signalling via MERCS can increase mitochondrial calcium import, metabolism and dynamics, while maladaptive UPR signalling can result in excessive calcium import and activation of apoptotic pathways. Targeting UPR signalling and the assembly of MERCS is an attractive therapeutic approach for a range of age-related conditions such as neurodegeneration and sarcopenia. This review highlights the emerging evidence related to the role of redox mediated UPR activation in orchestrating inter-organelle communication between the ER and mitochondria, and ultimately the determination of cell function and fate.

## Introduction

Disruption of organelle communication plays a pivotal role in the altered cellular homeostasis in older organisms and during disease progression. The cellular response to perturbations within the intracellular environment can be an adaptive and ultimately beneficial response, or a hormesis effect, where low levels of stress renders cells resistant to a subsequent challenge [1]. The beneficial hormesis effect is often preceded by an acute change in the cellular environment, such as in skeletal muscle during exercise where there is a site-specific increase in ROS that activates specific signalling pathways, such as Nrf2 activation [2, 3]. Chronic changes in the intracellular redox environment, result in maladaptive responses that can be detrimental and often described in pathological conditions and age-related diseases [4]. Cellular homeostasis is maintained by a constant flow of information from the external environment but also critically by inter-organelle communication, facilitating the exchange of material and information in response to biological perturbations. The endoplasmic reticulum (ER) and mitochondria are key regulatory hubs in maintaining cell homeostasis and they have a synergistic relationship that can determine their function and response to the cellular environment. Mitochondrial-ER contact sites (MERCS) mediate the exchange of information between these organelles and help determine how the cell responds to disruption in the cellular environment. The regulation of the assembly and disassembly of MERCS is an active area of research, in particular in the context of how MERCS change during development, age and disease and with subsequent effects on the function of both the ER and mitochondria.

## Endoplasmic reticulum stress and the unfolded protein response

The endoplasmic reticulum (ER) is the largest of the cell's membrane-bound organelles (~ 10% cell volume), it is composed of a continuous network of tubules and sacs surrounded by membranes or cisternae [5]. The ER contributes to proteostasis by regulating protein synthesis, folding and transport [6]. It is the main intracellular store of calcium (Ca^2+^), the ER releases Ca^2+^ into the cytosol in response to cellular signals, initiating a signalling cascade that can modulate a wide range of cellular functions [7]. The rough ER is composed of sacs with a high density of ribosomes attached to the cytosolic domain and involved in protein biosynthesis, while the smooth ER contains tubules that specialise in lipid synthesis [5, 8].

Protein folding is a key regulatory step in proteostasis and disruption can result in the accumulation of misfolded proteins. The ER has a unique environment that facilitates protein folding, its oxidising nature favouring the formation of disulphide bonds [6]. ER homeostasis can be altered by physiological and pathological conditions, leading to an accumulation of misfolded proteins in the ER lumen, referred to as ER stress and results in the activation of the unfolded protein response (UPR) [9]. A variety of cellular stress conditions can alter ER proteostasis, including disruption of Ca^2+^ homeostasis, protein glycosylation, redox imbalance and an accumulation of misfolded proteins [10]. The adaptive UPR^ER^ aims to restore proteostasis and alleviate ER stress by reducing protein translation, increasing the chaperone capacity of the ER and stimulating the degradation of misfolded proteins [6, 9].

### UPR activation

**Fig. 1:**
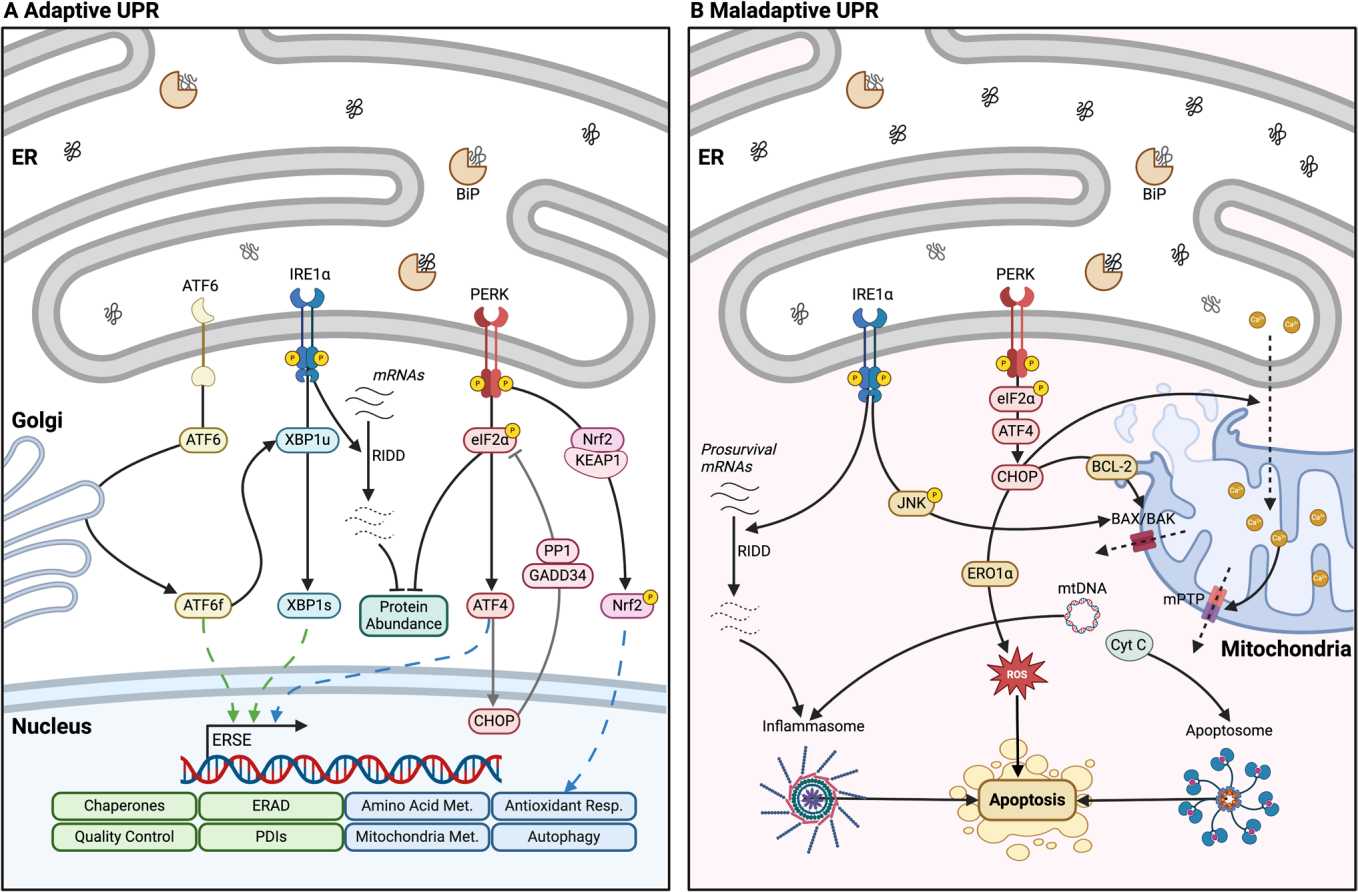
**The UPR**^**ER**^**. A** Adaptive **UPR**^**ER**^. Following ER stress, BiP binds to misfolded proteins on the substrate-binding site and the ATPase domain dissociates from the transmembrane receptors, allowing allosteric activation of the **UPR**^**ER**^ regulators by oligomerisation and phosphorylation [14, 15]. (1) IRE1α RNase activity mediates unconventional splicing of XBP1 [16–18], XBP1s translocates to the nucleus to promote expression of genes related to quality control [9, 19]. IRE1α also mediates the cleavage and degradation of mRNAs and microRNAs; regulated IRE1α-dependent decay (RIDD), decreasing the protein load in the ER lumen [20]. (2) PERK phosphorylates eIF2α [21], promoting rapid attenuation of global mRNA translation [22, 23]. Phosphorylated eIF2α also regulates the translation of the transcription factor ATF4 [24]. ATF4 regulates the feedback loop responsible for the restoration of protein synthesis. ATF4 induction of CHOP, upregulates the expression of GADD34 which forms a complex with PP1 to dephosphorylate eIF2α [25, 26]. (3) ATF6α translocates to the Golgi apparatus, where it is cleaved to generate ATF6f, which acts as a transcription factor that promotes the expression of ER chaperones [27, 28]. ATF6α promotes the expression of Xbp1 mRNA, enhancing the substrate load for IRE1α splicing [29]. **B** Maladaptive **UPR**^**ER**^. Following prolonged ER stress the homeostatic capacity of the **UPR**^**ER**^ becomes saturated that can activate pro-apoptotic signalling. (1) IRE1α interacts with TRAF2 to promote a kinase signalling cascade that activates JNK [30, 31]. JNK promotes the oligomerisation of BAX and BAK on the mitochondrial membrane and the assembly of the apoptosome [32, 33]. RIDD can promote apoptosis by degrading essential cell-survival mRNAs such as the negative regulators of TXNIP, promoting the assembly of the inflammasome leading to apoptosis [34, 35]. (2) PERK-eIF2α induces the translation of ATF4, activation of CHOP and GADD34 [25, 26]. CHOP promotes the expression of PUMA, NOXA, BIM and BID, which induce the mitochondrial BCL-2 pro-apoptotic proteins. CHOP can also activate the translation of ERO1α, promoting the oxidation of the ER environment [36, 37]. PERK-ATF4-CHOP arm regulates IP3R-mediated Ca^2+^ leakage from the ER [38, 39]. Sustained and excessive Ca^2+^ transport from the ER to the mitochondria impairs mitochondrial metabolism and lead to opening of the mPTP and pro-apoptotic signalling [40, 41]

The UPR^ER^ comprises three branches: inositol-requiring enzyme 1α (IRE1α), protein kinase RNA-like ER kinase (PERK) and activating transcription factor 6 (ATF6) [9]. These ER signalling proteins have a similar structure, consisting of ER luminal and cytosolic domains. The ER luminal domains are formed by a single pass through the membrane [9], while cytosolic domains are the mediators of the UPR^ER^ [9, 11]. Under physiological conditions, the chaperone BiP/glucose-regulated protein 78 (GRP78), binds to the luminal domains of the mediators of the UPR^ER^, repressing their activation [12, 13]. Upon accumulation of excessive unfolded or misfolded proteins in the ER lumen, BiP binds to misfolded proteins on the substrate-binding site and the ATPase domain dissociates from the transmembrane receptors, allowing allosteric activation of the UPR^ER^ regulators by oligomerisation [14, 15] (Fig. 1a).

### UPR^ER^ signalling

IRE1α is the most conserved signalling branch of the UPR^ER^, it is a type I transmembrane protein with Ser/Thr protein kinase and endoribonuclease activities [42]. Upon accumulation of misfolded proteins, BiP dissociates from IRE1α, inducing its oligomerisation and autophosphorylation [43, 44]. Phosphorylated IRE1α RNase activity mediates the unconventional splicing of an intronic region of XBP1 in the cytoplasm independently from the spliceosome, generating the active form, spliced XBP1 (XBP1s) [16–18] (Fig. 1a). XBP1s contains a basic leucine zipper domain (bZIP), it can translocate to the nucleus to induce expression of ER stress-response elements (ERSE), related to quality control (protein folding, translocation, and degradation) [9, 19]. IRE1α also mediates the cleavage and degradation of mRNAs and microRNAs; in a process known as regulated IRE1α-dependent decay (RIDD), decreasing the abundance of some mRNAs and reducing the protein load in the ER lumen [20]. IRE1α regulation of mRNAs and microRNAs depends on the presence of an IRE1α cleavage site formed by a stem-loop containing the sequence “CUGCAG” [45]. IRE1α has been demonstrated to degrade miR-17, -34a, -96, and -125b, these microRNAs target mRNA encoding the pro-apoptotic protein caspase-2, increasing the levels of this protein and initiating activation of apoptosis [46]. Furthermore, the cytosolic domain of IRE1α can interact with adapter proteins to establish crosstalk with other stress-mediator pathways [47]. The interaction of IRE1α with TRAF2 (tumour necrosis factor receptor (TNFR)-associated factor-2) promotes the activation of ASK1/JNK [30], ERK and p38 [48], protein kinases involved in autophagy, apoptosis and NF-κB inflammatory pathways [49].

PERK is a type I protein kinase that dissociates from BiP under ER stress, it is activated by dimerization and autophosphorylation [12]. Active PERK phosphorylates eIF2α at serine 51 [21], promoting a rapid attenuation of global mRNA translation, reducing the protein load for folding in the ER [22, 23]. Phosphorylated eIF2α also controls the selective translation of the transcription factor ATF4 [24] (Fig. 1a). ATF4 promotes the translation of ER stress genes related to the restoration of cellular homeostasis: protein synthesis, amino acid metabolism, redox homeostasis, apoptosis and autophagy [9]. ATF4 orchestrates the restoration of protein synthesis when the ER stress levels have been reestablished by regulating a feedback loop responsible for eIF2α dephosphorylation. The feedback loop is mediated by the induction of C/EBP homologous protein (CHOP) by ATF4, upregulation of GADD34 (growth arrest and DNA damage 34), which forms a complex with PP1 (a serine/threonine-protein phosphatase) to dephosphorylate eIF2α [25, 26].

ATF6α is a type II transmembrane protein that possesses a cytosolic N-terminus containing a bZIP motif [50]. ATF6α is located on the ER membrane with BiP bound to its Golgi localisation sequences. Under ER stress BiP is released from ATF6α, allowing translocation to the Golgi apparatus [51]. In the Golgi apparatus ATF6α is cleaved by Site-1 and 2 proteases (S1P and S2P), generating the N-terminal cytoplasmic fragment (ATF6f) containing the bZIP motif [27, 28] (Fig. 1a). ATF6f, following translocation to the nucleus, promotes the expression of ERSE and the ER chaperones (BiP and GRP94), affecting protein folding, maturation, translocation, and degradation [27, 28]. ATF6f and IRE1α constitute a regulatory hub of signalling pathways that are normally activated simultaneously for the regulation of XBP1s [29]. ATF6α promotes the expression of XBP1 mRNA, enhancing the substrate load for IRE1α splicing [29]. ATF6α also heterodimerises with XBP1s for the transcription of genes required for ER associated degradation (ERAD). Finally, XBP1s and ATF6f promote cellular secretory capacity by inducing the expansion of the ER and Golgi apparatus [52–54].

ERAD is activated alongside the UPR^ER^ [10, 55]. ERAD involves the recognition of misfolded proteins in the ER, their retrotranslocation to the cytoplasm, ubiquitination and subsequent degradation by the proteasome [55]. The induction of ERAD is regulated by the UPR^ER^, although there is crosstalk between these two mechanisms as ERAD can coordinate the expression of IRE1α [56].

### Adaptive UPR^ER^ signalling

The regulation and activation of the UPR^ER^ is dose-dependant, a low dose of an ER stressor can activate adaptive UPR^ER^, while in response to higher doses or chronic ER stress, maladaptive UPR^ER^ is induced [57] (Fig. 1). Adaptive UPR^ER^ activation (Fig. 1a) can promote an increase in the translation of chaperones, Ca^2+^ binding proteins and activation of antiapoptotic and antioxidant signalling pathways [58–60]. Ageing is associated with an alteration of ER morphology and the expression levels of ER chaperones and transducers, resulting in an impairment of the adaptive UPR^ER^ [59]. Subsequently cells are more susceptible to alterations in proteostasis and the ability to adapt to disrupted homeostasis [61].

Adaptive UPR^ER^ has been linked to a signalling network that improves the ageing phenotype. The stage of life of the organism, whether during development or maturity, can determine the hormesis effect of activation of the UPR^ER^ which is related to the decline in the inducibility of these pathways with age [62]. In *C. elegans* it was demonstrated that the inducibility of the UPR^ER^ peaks in the early developmental stages and declines in adulthood [62]. Exposure of *C. elegans* during larval development to low doses of tunicamycin (0.125 µg/ml) for 24 h resulted in increased lifespan and animals that had a delayed age-associated reduction in inducible UPR^ER^ activation [63]. Activation of IRE1-XBP1 arm can improve organismal development, stress resistance, and longevity [63–66]. During dietary restriction in *C. elegans*, the IRE1-XBP1 arm activates ERAD and results in increased longevity [63]. Similarly in *C. elegans*, it was demonstrated that expression of XBP1s in neurons, led to extended lifespan by triggering an adaptive UPR^ER^ in distant non-neuronal cells [65]. Activation of the ATF4 signalling pathway has also been demonstrated to extend lifespan in *C. elegans* [67, 68] and *Saccharomyces cerevisiae* [69].

### Maladaptive UPR^ER^ signalling

Following prolonged ER stress, the homeostatic capacity of the UPR^ER^ becomes saturated and results in pro-apoptotic signalling, regulated by IRE1α and PERK, with increased Ca^2+^ release from the ER (Fig. 1b). Under prolonged ER stress phosphorylated IRE1α interacts with TRAF2 to promote a kinase signalling cascade that ultimately activates JNK (Jun amino-terminal kinase) [30, 31]. JNK can promote apoptosis through activation of the mitochondrial BCL-2 pro-apoptotic proteins, BAX and BAK [32]. Oligomerisation of BAX and BAK promotes the assembly of the apoptosome [33]. Activation of the RIDD pathway by IRE1α can promote apoptosis by degrading essential cell-survival mRNAs such as chaperone BiP [70]. RIDD can degrade microRNAs that negatively target the expression of caspase 2, mediating BAX/BAK dependant apoptosis [46]. Finally, RIDD is involved in the degradation of negative regulators of thioredoxin-interacting protein (TXNIP), promoting the assembly of the inflammasome leading to apoptosis [34, 35] (Fig. 1b).

The PERK-eIF2α branch of the UPR^ER^ induces the translation of ATF4, activation of CHOP and GADD34 [25, 26]. CHOP regulates mitochondrial BCL-2 pro-apoptotic proteins, BAX and BAK through upstream regulators such as BH1-3 pro-apoptotic proteins; PUMA, NOXA [71], BIM [72] and BID [73, 74]. Activation of GADD34 by CHOP can restore protein translation in homeostatic conditions, however when proteostasis is not recovered, they can disrupt oxidative folding and result in altered ROS generation in the ER lumen [36, 75]. In addition, CHOP can activate the translation of ERO1α, involved in the formation of disulphide bonds in nascent proteins, but during ER stress promotes oxidation of the ER environment [36, 37]. The disruption of the ER redox state promotes leakage of H_2_O_2_ to the cytoplasm that can further induce apoptotic signalling [36] (Fig. 1b).

### Redox regulation of the ER

The intracellular redox environment is closely linked to the initiation of ER stress and UPR^ER^ activation. For example the disulphide reducing agent, dithiothreitol, is commonly used as an inducer of ER stress, as it can interfere with the redox dependent protein folding mechanisms within the ER [76]. In response to both endogenous and external stressors, the ER increases its protein folding capacity and activates defence mechanisms, such as autophagy and the antioxidant response [77, 78]. In the ER there is a constitutive production of H_2_O_2_ as a biproduct of oxidative protein folding, that promotes the formation of covalent disulphide bonds on nascent polypeptide chains [76]. The ER has a more oxidising environment compared to the cytosol that facilitates thiol disulphide exchange for correct protein folding and the ratio of GSH/GSSG is much lower compared to other organelles [79]. Oxidative folding is catalysed by ER-resident protein disulphide isomerases (PDIs), endoplasmic reticulum protein 72 (Erp72) and endoplasmic reticulum 57 (Erp57) [80]. The Cys residues located in the active site of PDI’s are reduced upon oxidation of the polypeptide, promoting the formation of disulphide bonds and subsequently re-oxidised by ER oxidoreductase 1 (ERO1) [81, 82]. ERO1 can transfer electrons to molecular oxygen (O_2_) and as a result generate H_2_O_2_, constituting a basal source of ROS in the ER [83]. Correct oxidative folding of proteins is essential for maintaining ER homeostasis, as impairment can induce the accumulation of both unfolded proteins and ROS in the lumen of the ER [76]. In *C. elegans* it was demonstrated that during ageing there is a shift in the redox state of the ER to more reducing conditions compared to the cytosol, which becomes more oxidised with age [84]. As a result of reduced folding capacity within the ER, cells are more sensitive to maladaptive UPR signalling or ER stress response failure, as described in metabolic disease and ageing [85]. Organelle specific changes in the redox environment reflect the distinct functions of these organelles and how redox homeostasis within different compartments needs to be regulated. Table 1 contains ER and MERCS localised proteins identified with redox specific post transcriptional modifications.

**Table 1:** Proteins identified with redox post-translational modifications (PTMs) involved in ER-mitochondria communication

Protein	Subcellular localization	Biological process	Redox related PTMs	References
ACO	Mitochondria	TCA cycle	Redox regulation of Cys residues in regulating Fe-S clusters	[271]
AKT	Mitochondria	Mit. Survival	Disulphide between Cys297 and Cys311	[272]
ANT	Mitochondria	mPTP	Disulphide between Cys160 and Cys257	[273]
ATF6	ER	UPR^ER^	Inter and intramolecular disulphides in luminal domain (Cys467 and Cys618)	[274]
ATF6	ER	UPR^ER^	ATF6α disulphide reduction during ER stress, by PDIA5	[87]
CAC	Mitochondria	TCA cycle	Glutathionylation of Cys136 and Cys155	[275]
Complex I, 75-kDa subunit	Mitochondria	ETC	Glutathionylation of Cys531 and Cys704	[276]
Complex I, ND3	Mitochondria	ETC	Functional redox switch Cys39 exposed in inactive state	[277]
Complex II, 70-kDa subunit	Mitochondria	ETC, TCA cycle	Redox regulation of Cys90, by S-glutathionylation	[278]
Complex V, α-subunit	Mitochondria	ETC	Functional redox regulation by of Cys294	[279]
Complex V, α-subunit and γ-subunit	Mitochondria	ETC	Functional redox regulation by disulphide bond between Cys294 and Cys103	[279]
CYP-D	Mitochondria	mPTP	Functional redox regulation of Cys203	[280]
DNAJA1	Cytosol	UPR^mt^	Redox modifications of Cys149 and Cys150	[281]
DRP1	Mitochondria, MAMs	Mit. dynamics	Functional redox regulation of Cys644	[282]
ERO1α	ER, MAMs	Oxidative folding, MERCS	Cys94-Cys99 disulphide bond	[283]
ERO1β	ER	Oxidative folding	Cys90-Cys95 disulphide bond	[283]
ERp72	ER	Oxidative folding	Cys-X-X-Cys motif in catalytic site	[284]
ERp57	ER	Oxidative folding	Cys-X-X-Cys motif in catalytic site	[284]
IRE1α	ER	UPR^ER^	Oxidation of conserved Cys605, Cys630, Cys715 and Cys951	[91, 285]
IRE1α	ER	UPR^ER^	IRE1α Cys148 and Cys332 involved in disulphide bonds	[286]
IRE1α	ER	UPR^ER^	Disulphide between IRE1α Cys148 & PDIA6 Cys residue, regulates IRE1α dephosphorylation	[86]
GPx7	ER	Oxidative folding	Peroxidatic Cys57 and Resolving CysCys87	[287]
GPx8	ER	Oxidative folding	Peroxidatic Cys79 and Resolving Cys108	[288]
GRP78	ER	UPR^ER^	GPx7 activation by disulphide bond Cys41-Cys420	[289]
IDH	Mitochondria	TCA cycle	Inactivation by glutathionylation of Cys269	[290]
IP_3_R1	ER, MAMs	Ca^2+^ Signalling, MERCS	Functional redox regulation of Cys206 and Cys214 (cytosolic suppressor domain), Cys1394 and 5 basally oxidised Cys	[291]
MCU	Mitochondria, MAMs	Ca^2+^ Signalling, MERCS	Redox regulation of Cys97	[292]
MFN1	Mitochondria, MAMs	Mit. dynamics, MERCS	Redox regulation by disulphide bond between MFN1 and MFN2-Cys684	[293]
MFN2	Mitochondria, MAMs	Mit. dynamics, MERCS	Redox regulation by disulphide bond between MFN1 and MFN2-Cys684	[293]
MID49/51	Mitochondria, MAMs	Mit. dynamics, MERCS	Functional redox regulation by oligomerisation	[294]
ODH	Mitochondria	TCA cycle	Functional redox regulation by sulfenylation, sulfinylation, and S-glutathionylation	[295]
PDH	Mitochondria	TCA cycle	Functional redox regulation of Cys residue	[296]
PDI	ER	Oxidative folding	Cys-X-X-Cys motif in catalytic site	[284]
PERK	ER, MAMs	UPR^ER^, MERCS	PDIA6, PDI and ERp57 are involved in the redox regulation of PERK, likely involves disulphide bond formation	[86, 89]
PERK	ER, MAMs	MERCS	Redox regulation of PERK-ERO1⍺ in MAMs requires PERK Cys216	[90]
PRDX3	Mitochondria	Antioxidant Response	Peroxidatic Cys47 and Resolving Cys168	[297]
PRDX4	ER	Oxidative folding	Peroxidatic Cys127 and Resolving Cys248	[298]
PRDX5	Mitochondria	Antioxidant Response	Peroxidatic Cys48 and Resolving Cys152	[299]
PTEN	Mitochondria	Mitophagy	Disulphide bond Cys71-Cys124	[300]
QSOX	ER	Oxidative folding	Cys-X-X-Cys motif in the catalytic site	[301]
RyR1	ER, MAMs	Ca^2+^ Signalling, MERCS	Functional redox regulation of Cys253, Cys1040, and Cy1303 and others endogenously modified	[302]
SERCA	ER	Ca^2+^ Signalling	Functional redox regulation of Cys674	[303]
VDAC1	Mitochondria, MAMs	Ca^2+^ Signalling, MERCS	Redox sensitive Cys127 & Cys232	[304]
VKOR	ER	Oxidative folding	Cys-X-X-Cys motif in catalytic site	[305]

*ACO* Aconitase, *B-AKT* protein kinase, *ANT* adenine nucleotide translocator, *6-ATF6* activating transcription factor, *CAC* carnitine/acylcarnitine carrier, *Complex I, 75-kDa subunit; Complex I, ND3; Complex II, 70-kDa subunit; Complex V, α-subunit; Complex V, γ-subunit; *D-CYP-D, cyclophilin, DNAJA1 DnaJ hsp40 family member A1, *DRP1* dynamin-related protein 1, *ERO1a* endoplasmic reticulum oxidoreductase 1 alpha, *ERO1b* endoplasmic reticulum oxidoreductase 1 beta, *ERp72 *protein disulphide isomerase family A, member 4; *ERp57 *protein disulphide isomerase family A, member 3, *IRE1a* inositol-requiring enzyme type 1 alpha, *7-GPx7 *glutathione peroxidase, *8-GPx8 *glutathione peroxidase, *78-GRP78 *glucose-regulated protein, *IDH* isocitrate dehydrogenase, *IP3R1 *inositol 1,4,5-trisphosphate receptor type1, *MCU* mitocondrial calcium uniporter, *1-MFN1 *mitofusin; *2-MFN2 *mitofusin, *MID49/51 *mitochondrial dynamics protein49/51, *ODH* 2-oxoglutarate dehydrogenase, *PDH* pyruvate dehydrogenase, *PDI* protein disulfide isomerase, *PERK* protein kinase RNA-like ER kinase, *3-PRDX3 *peroxiredoxin, *4-PRDX4* peroxiredoxin, *5-PRDX5* peroxiredoxin, *PTEN* phosphatase and tensin homolog, *QSOX* quiescin sulfhydryl oxidase, *RyR* ryanodine receptors, *SERCA* sarco/endoplasmic reticulum Ca^2+^ ATPase, *1-VDAC1*, voltage-dependent anion-selective channel; *VKOR* vitamin K epoxide reductase

The UPR^ER^ response can also be activated by alterations in the redox state of the ER. Activation of the UPR^ER^ in response to oxidative stress is mediated by two members of the PDIs, PDIA5 and PDIA6, which facilitate thiol–disulphide exchange on Cys residues of the luminal domains of IRE1, PERK and ATF6 [86, 87]. ER generated ROS can induce ATF6 signalling, PDIA5 cleaves disulphide bonds in ATF6, promoting oligomer dissociation and translocation from the ER to Golgi and expression of ATF6 target genes [87]. ROS can also activate IRE1α and PERK signalling, when PDIA6 binds to the luminal domain of both UPR^ER^ sensors and promotes thiol disulphide exchange, similar to ATF6 activation [86, 88, 89]. Following initial ER stress PERK Cys216 can be reversibly oxidised allowing formation of covalent interactions with ERO1α, resulting in a tightening of MERCS formation and increased Ca^2+^ flux into mitochondria and regulating mitochondrial bioenergetics [90]. IRE1α also provides a metabolic link between UPR^ER^, redox signalling and mitochondrial function. Sulfenylation of a conserved Cys residue located in the IRE1α kinase loop can inhibit its kinase activity and promote p38 activation of the Nrf2/SKN-1 dependent antioxidant response, regulating cytoplasmic ROS and inhibiting the UPR [91]. IRE1α therefore lies at a metabolic hub that dictates cell fate via activation of the UPR, initiation of the antioxidant response (via Nrf2 activation) or activation of apoptotic cell death via enhanced Ca^2+^ entry into mitochondria.

### Calcium signalling in the ER

The ER is the main Ca^2+^ store in metazoan cells, regulating Ca^2+^ homeostasis which is vital for cellular function. In the lumen of the ER, chaperones including calreticulin, calnexin, BiP, GRP94 and PDI, maintain Ca^2+^ levels within a physiological range [92]. Many of these chaperones are implicated in ER stress and ROS sensing, connecting these responses with Ca^2+^ homeostasis [76]. Ca^2+^ flux within the ER is mediated by sarco/endoplasmic reticulum Ca^2+^ transport ATPase (SERCA) family, which regulates the pumping of Ca^2+^ inside the ER in an ATP-dependent process [93]. Thapsigargin, an inhibitor of SERCA and commonly used to promote the induction of ER stress. The release of Ca^2+^ to the cytosol is controlled by the inositol 1,4,5-trisphosphate receptor (IP3R) and the ryanodine receptor (RyR) families [94, 95].

Under ER stress conditions a decrease in ER Ca^2+^ levels has been associated with the inhibition of SERCA activity [96–98] and passive leak of Ca^2+^ from the ER due to altered IP3R activity [99]. An increase in ROS within the ER has been demonstrated to promote the release of Ca^2+^ from the ER, linked with oxidation of specific Cys residues of Ca^2+^ regulators including SERCA [100], IP3R [101] and RyR [102]. Perturbations in Ca^2+^ homeostasis within the ER will inhibit the function of Ca^2+^-dependent ER chaperones potentially resulting in ER stress [103]. Ca^2+^ flux between the ER, cytoplasm and mitochondria can also determine apoptotic signalling during prolonged ER stress [104]. The PERK-ATF4-CHOP arm regulates Ca^2+^ flux by CHOP induction of ERO1α, that subsequently induces IP_3_R-mediated Ca^2+^ leakage from the ER [38, 39]. Sustained ER stress and excessive Ca^2+^ transport from the ER to the mitochondria can impair mitochondrial metabolism and lead to opening of the mitochondrial membrane permeability transition pore (mPTP) and pro-apoptotic signalling [40, 41] (Fig. 1b). Ca^2+^ release into the cytoplasm also activates Calpain proteases, which cleaves and activates caspase 12, triggering the induction of apoptosis [105, 106].

### Mitochondria

Mitochondria are essential organelles with multi-faceted functions including energy generation via oxidative phosphorylation, iron metabolism, ion and phospholipid homeostasis. Mitochondria are also involved in the generation of ROS and subsequent redox signalling, Ca^2+^ homeostasis, apoptosis and autophagy. Disruption of mitochondrial function has been implicated in almost all age-related diseases including sarcopenia, neurodegeneration and cancer [107]. Mitochondria are in constant dynamic flux determined by the balance between biogenesis, mitochondrial fusion and fission along with selective degradation via mitophagy [107]. Mitochondrial morphology has been linked to substrate use, with fragmented mitochondria demonstrating increased fatty acid oxidation, linking mitochondrial dynamics and cellular fuel preference [108]. Indeed mitochondrial morphology can change rapidly in response to metabolic demand during exercise [109] or in proliferating cells such as in stem cells or cancerous cells, where mitochondrial fission predominates over fusion and is characterised by a fragmented mitochondrial network [110].

Mitochondria and ER are linked at MERCS facilitating the dynamic flow of information between the organelles, allowing changes in ER homeostasis to regulate mitochondrial function [111, 112]. Early adaptive ER stress promotes the formation of contact sites and facilitates Ca^2+^ transfer to mitochondria that increases mitochondrial metabolism [113], increasing energetics to alleviate ER stress [114].

### Mitochondrial dynamics

Mitochondrial biogenesis is a complex process requiring the integration of mitochondrial DNA, lipids and proteins, responding to stimuli such as hypoxia and metabolic demand [115]. Mitochondrial division stimulates the recruitment of proteins and components to existing mitochondrial compartments and complexes, ensuring that biogenesis is closely coupled to mitochondrial fusion and fission [116]. The regulation of mitochondrial degradation via mitophagy is controlled by a number of pathways including: Ubiquitin dependent degradation via the Pink/Parkin pathway, receptor mediated mitophagy via BNIP3, BNIP3L/NIX and FUNDC1, that facilitate direct interaction with the autophagosome [117, 118]. AMPK mediated mitophagy has also been described in conditions of high metabolic demand, with AMPK interacting antagonistically with mTORC1 to promote mitophagy [119]. The precise mechanisms underlying basal levels of mitochondrial degradation or in response to acute and chronic stress are still to be defined, although it is increasingly recognised that MERCS play a key role in determining mitochondrial dynamics [112, 120, 121]. Key regulators of mitochondrial biogenesis and turnover such as PGC1α, DRP1, MFN2 and OPA1 have been demonstrated to be regulated by the redox environment [122, 123].

### Mitochondrial stress sensing

**Fig. 2:**
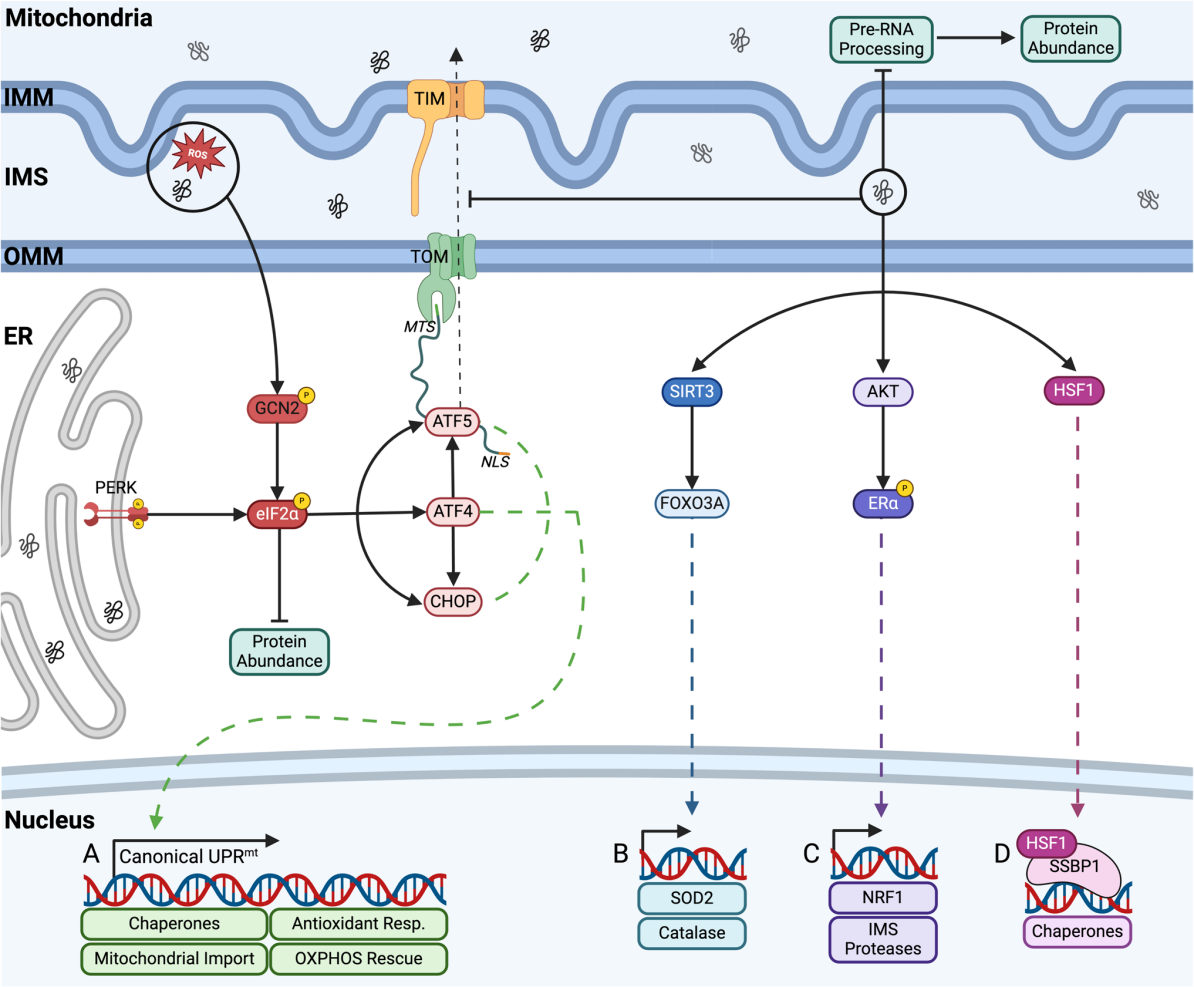
**The UPR**^**mt**^. Most proteins that constitute the mitochondrial proteome are synthesised in the cytoplasm, targeted and imported into mitochondria [125] via the TOM/TIM complex [126], perturbation of this trafficking can impair mitochondrial proteostasis and induce mitochondrial stress [127]. **A** The canonical axis of the UPR^mt^ is controlled by the expression of ATF5, ATF4 and CHOP [132]. CHOP alleviates proteotoxic stress by inducing the expression of the mitochondrial chaperones HSP10 and HSP60 [134]. ATF5 is normally imported into mitochondria via TOM and TIM, where it is degraded by proteases [138]. Mitochondrial proteotoxic stress will perturb mitochondrial import efficiency, resulting in the activation of ATF5 by p-eIF2α and its translocation to the nucleus [139]. ATF5 promotes the transcription of genes related to chaperones, proteases and antioxidant proteins [137]. **B** The sirtuin axis of the UPR^mt^ activates SIRT3, which deacetylates FOXO3A, promoting its translocation to the nucleus and transcription of SOD2 and catalase [142, 143]. **C** AKT mediates the ROS-dependant phosphorylation of ERα, which activates NRF1 and the IMS protease HTRA2 [144]. NRF1 stimulates mitochondrial respiration, proteasome activity and the IMS protease OMI. **D** Mitochondrial proteotoxic stress promotes epigenetic changes in the cellular DNA regulated by HSF1, it translocates to the nucleus where it interacts with SSBP1 to bind to the chromatin and boost the expression of mitochondrial chaperones [145, 146]

Mitochondrial DNA (mtDNA) contains 37 genes, of which 13 encode structural polypeptides of components of electron transport chain (ETC) complexes [124]. Most proteins that constitute the mitochondrial proteome are synthesised in the cytoplasm, targeted and imported into mitochondria, where they bind to mitochondrial-localised chaperones to help their translocalisation and assembly [125]. Trafficking of proteins into the mitochondrial matrix via the TOM/TIM complex (translocase of the outer membrane/translocase of the inner membrane) [126] needs to be carefully controlled since disruption could impair mitochondrial proteostasis and overwhelm the chaperone capacity within mitochondria, inducing mitochondrial stress [127] (Fig. 2). Any perturbation of mitochondrial proteostasis that induces mitochondrial stress, activates pathways related to the integrated stress response (ISR) [128]. The ISR is activated to restore homeostasis in response to various types of stress conditions and ultimately results in the phosphorylation of eIF2α Ser51 [129]. Phosphorylated eIF2α activates ATF4, inducing the attenuation of protein translation and promoting the expression of mRNAs encoding CHOP and ATF4, which promotes expression of ATF5 [130, 131].

### Mitochondrial UPR

The canonical axis of the UPR^mt^ is controlled by the expression of ATF4, ATF5 and CHOP, three bZIP transcription factors central to the ISR [132]. ATF4 promotes the expression of genes related to the UPR^mt^, however it mainly acts as a regulator of both ATF5 and CHOP expression [133]. CHOP alleviates proteotoxic stress by inducing the expression of the mitochondrial chaperones HSP10 and HSP60 [134]. CHOP has been also proposed as a regulator of the protease complex ClpXP, which plays a key role in sensing and maintaining proteostasis (through the ClpP proteolytic subunit) inside the mitochondrial matrix [135]. ClpXP has been reported to activate UPR^mt^ under conditions of mitochondrial proteotoxic stress [136]. ATF5 possesses a mitochondrial-targeting sequence (MTS) and a nuclear localisation sequence (NLS) [137]. Under homeostatic conditions, ATF5 is imported into healthy mitochondria via TOM and TIM, where it is degraded by proteases, thus acting as a sensor of mitochondrial import efficiency [138]. However, under overload of misfolded proteins, protein aggregation and perturbed mitochondrial import efficiency, ATF5 is activated by p-eIF2α and translocated to the nucleus, where it increases folding capacity via retrograde signalling [139] (Fig. 2a). ATF5 promotes the transcription of genes that aid in the recovery of normal proteostasis, for example by upregulating chaperonins, chaperones, proteases and antioxidant proteins [137]. Impaired mitochondrial protein import efficiency results in the accumulation of mistargeted mitochondrial proteins in the cytosol, that will activate the UPR^am^ (UPR activated by mistargeted proteins), which enhances the assembly of the proteasome in order to degrade potentially toxic mislocalised proteins [140, 141].

The sirtuin axis of the UPR^mt^ boosts the antioxidant capacity of the cell in response to disrupted proteostasis, driven by the increase in mitochondrial ROS derived from mitochondrial dysfunction and activation of the canonical UPR^mt^ [131]. During mitochondrial proteotoxic stress, activation of SIRT3 results in deacetylation of FOXO3A, promoting its translocation to the nucleus and transcription of SOD2 and catalase [142, 143] (Fig. 2b). Under proteotoxic stress in the IMS, AKT mediates the ROS-dependant phosphorylation of ERα, which increases the expression of nuclear respiratory factor 1 (NRF1) and the IMS protease HTRA2 transcripts [144]. NRF1 mediates the activation of protein quality control by stimulating mitochondrial respiration [147], proteasome activity and the expression of the IMS protease OMI [144] (Fig. 2c). Mitochondrial proteotoxic stress also promotes epigenetic changes, through the induction of chromatin remodelling factors that facilitate the induction of mitochondrial chaperones [145]. These changes are regulated by HSF1, which also plays a key role in the heat-shock response and forms a complex with mitochondrial single-stranded DNA binding protein 1 (SSBP1) [145, 146]. HSF1 translocates to the nucleus where it binds to the chromatin remodelling factor BRG1 and completes the formation of the chromatin remodelling complex, which will ultimately increase the expression of chaperones to protect mitochondrial function [146] (Fig. 2d).

Acute mitochondrial stress activates the translation axis of the UPR^mt^, leading to a decrease in pre-RNA processed product and decreased mitochondrial translation, reducing the folding load in mitochondria [148]. This axis of the UPR^mt^ works as a first defence mechanism against proteotoxic stress, it is activated in stressed mitochondria before the activation of the canonical UPR^mt^ [148] (Fig. 2d). mtDNA is transcribed into long pre-RNAs, processed by the RNase P complex (formed by MRPP1, 2 and 3). Activation of the translation axis of the UPR^mt^, reduces MRPP3 levels, as a result some of the mitochondrial long pre-RNAs are not translated with a subsequent reduction in mitochondrial protein biosynthesis [149, 150].

### Redox regulation of mitochondria

Mitochondrial respiration generates ATP but can also result in ROS generation, both superoxide and H_2_O_2_ as a result of electron leak from redox donors in the ETC, reducing molecular oxygen to superoxide and its subsequent conversion to H_2_O_2_ [151]. Mitochondrial ROS generation has been described at various sites along the ETC in particular at complex I and III by both forward and reverse electron transport as well as in conditions of hypoxia, indicating mitochondria are key regulatory hubs for redox signalling in cellular homeostasis and pathologies [151, 152]. ROS generation during reverse electron transport has been identified as a major cause of oxidative damage in conditions such as ischaemia where there is an accumulation of succinate [153]. Succinate is a substrate for the TCA enzyme succinate dehydrogenase at complex II, a FAD-dependent enzyme from the IMM that participates in the reduction of ubiquinone [152]. During conditions of mitochondrial hyperpolarisation, reverse electron transport results in electrons flowing back to complex I, generating NADH and superoxide [154]. Under controlled conditions where complex I and III are blocked and levels of succinate reduced, complex II has the capacity to generate significant levels of ROS both in forward (accepts electrons from succinate) and reverse (accepts electrons from ubiquinol) modes [155]. Cys39 of ND3 subunit of complex I has been identified as a critical redox switch in determining its catalytic active state, this Cys residue becomes accessible to alkylating agents in the inactive D-state [156]. Temporal reversible oxidation of Cys39 of ND3 has become a therapeutic target in ischaemia as when reversibly oxidised, complex I remains in an inactive state preventing reverse electron transport and subsequent superoxide generation [153]. In acute hypoxia, complex I acidifies the mitochondrial matrix which can solubilise Ca^2+^ and activate the Ca^2+^/Na^+^ antiporter, causing a decrease in IMM fluidity, this can result in a reduction in the diffusion rate of ubiquinol from complex II to complex III, promoting ROS generation [157]. ROS generation within mitochondria particularly the IMS has the capacity to result in redox modifications of sensitive proteins affecting their function and overall mitochondrial capacity. Redox modifications of proteins imported into the IMS can also affect mitochondrial activity as a result of disrupted assembly of complexes within the ETC due to the altered redox environment [158]. Table 1 contains mitochondrial localised proteins identified with redox specific post transcriptional modifications.

The mitochondrial redox environment also regulates mitochondrial dynamics, sites of mitochondrial fission have distinct ROS signatures, fission at the periphery or tip results in mitochondrial fragments destined for degradation while midzone fission is preferential for dynamics [159]. Disrupted mitophagy can result in an accumulation of dysfunctional mitochondria and has been associated with a range of age-related diseases particularly in tissues with high metabolic demand such as neurons and skeletal muscle [160, 161]. Chronic mitochondrial dysfunction leads to the accumulation of mitochondrial generated ROS, which can promote the unfolding/misfolding and aggregation of proteins inside the organelle and propagate mitochondrial dysfunction [162]. An increase in mitochondrial dysfunction can induce activation of the UPR^mt^, in particular ATF5 activation, in order to resolve proteotoxic and oxidative stress [137, 163]. In *C. elegans* it was demonstrated that the orthologue of ATF5, ATFS-1 has a dual action to protect cells from mitochondrial dysfunction, as it can upregulate genes involved in mitochondrial proteostasis (such as chaperones to restore protein homeostasis or glycolysis to boost ATP production) and bind promoters of NADH ubiquinone oxidoreductase assembly factors to maintain the function of the ETC complexes in order to optimise respiratory capacity during mitochondrial stress [164].

Low levels of mitochondrial stress can result in a mitohormesis response, the initial activation of stress signalling pathways that ultimately result in adaptive responses to improve stress resistance. A link between ROS production and mitohormesis has been repeatedly demonstrated in *C. elegans*, for example glucose deprivation resulted in enhanced respiration, increased ROS generation and extended the lifespan of the nematodes [165]. Inhibition of mitochondrial complex I with low doses of rotenone has also been demonstrated to promote lifespan extension in *C. elegans* [166]. The amount and duration of ROS generated by the ETC can influence lifespan and behaviour in model organisms [167, 168]. Similarly a recent study using *Drosophila* and mice pre-treated with N-acetyl-L-tyrosine, induced the production of ROS and promoted stress resistance related to mitohormesis [169].

Mitochondrial metabolism is modulated by Ca^2+^-dependent mechanisms linked to the ER stress response, through the stimulation of CHOP expression and phosphorylation of eIF2 and JNK [170]. The exchange of information via metabolites, ions and lipids between the ER and mitochondria can alter ATP production and promote reorganisation of the mitochondrial network [113]. Induction of an adaptive UPR^ER^ has been demonstrated to increase mitochondrial biogenesis, through the PERK-Nrf2 pathway [171]. ER stress can promote changes in the morphology of mitochondrial by promoting UPR^ER^ induced mitochondrial hyperfusion, in a process dependent on the phosphorylation of eIF2α by PERK [172]. A study in *Drosophila* demonstrated mitochondrial ETC disruption specifically activated PERK, while the other branches of the UPR^ER^ were not responsive [173]. This was attributed to PERK localisation at mitochondria-associated ER membranes (MAMs), making it more sensitive to respond to local stress signals [173].

In *C. elegans,* ATFS-1 regulates mitochondrial biogenesis and network expansion during normal development [174]. High levels of mitochondrial protein synthesis are needed during development, this results in a reduction in the levels of ATFS-1 imported into mitochondria. Subsequently ATFS-1 is translocated to the nucleus and results in the activation of the UPR^mt^, promoting the expansion of the mitochondrial network [174]. Mild mitochondrial stress can initiate a hormesis response that increases lifespan in *C. elegans*, this effect can activate the UPR^mt^ leading to descendants with higher levels of mtDNA that exhibit longer lifespans; increased resistance to infection, heat shock, and oxidative stress; although with slower development and lower fertility compared to those with normal mtDNA and UPR^mt^ levels [175]. ATFS-1 regulates the accumulation of transcripts of OXPHOS from both the nuclear and mitochondrial genomes in order that biogenesis of the ETC complex aligns with the ability of the stressed organelles to fold proteins and assemble ETC complexes [164].

### Mitochondrial ER contact sites

**Fig. 3:**
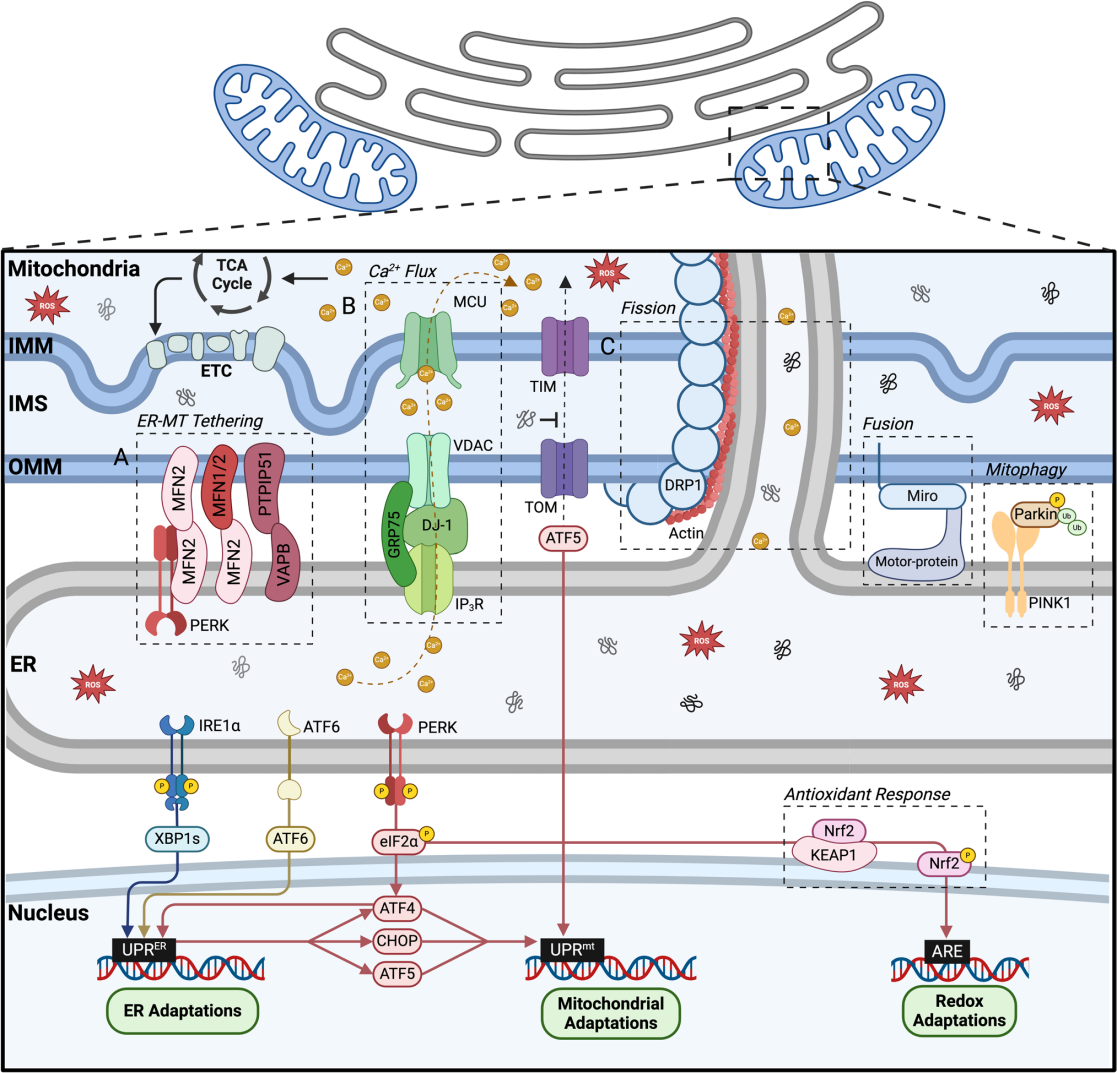
Mitochondria-ER contact sites molecular components and cellular functions. MERCS are relatively stable structures that require the formation of molecular bridges established by interacting proteins anchored in the smooth ER and the OMM [5]. Tethering complexes are essential, structural and reversible bonds that stabilise MERCS [177]. **A** MERCS tethering complexes occur between ER MFN2 and mitochondrial MFN2 or ER MFN2 and MFN1 [178]. The MFN tethering complex is dependent on the interaction of MFN2 and PERK at the ER membrane, essential for the establishment of the contact sites [90, 179]. Other complexes reported as regulating the tethering of MERCS include the ER VAPB and the OMM PTPIP51 [180]. **B** MERCS regulate Ca^2+^ flux between the ER and the mitochondria by the complex that forms between IP3R from the ER and VDAC from the OMM [5, 177]. Ca^2+^ passes through the MCU to reach the mitochondrial matrix [185, 186]. DJ-1 [187] and GRP75 [188] regulate the connection between IP3R and VDAC [189]. Some components of the TCA cycle require the binding of Ca^2+^ for their function, the interaction of mitochondria and ER via MERCS supply Ca^2+^ to mitochondria for stimulating the TCA cycle, resulting in an increase in ATP production [190]. **C** MERCS control the processes of mitochondrial fusion, fission and mitophagy [111, 191]. The ER promotes the polymerisation of actin filaments and establishment of close contacts between the two organelles [192]. ER tubules will release Ca^2+^ ions into the mitochondria, triggering the inner mitochondrial membrane to divide [192, 193]. DRP1 assembles around mitochondria at the fission site, a DRP1 ring constricts with the aid of actin–myosin filaments, resulting in the formation of two daughter mitochondria. ER tubules guide the position and timing of mitochondria fusion through the tethering with mitochondria [191, 194]. During mitochondrial fusion the contact sites between the tubules and the mitochondria need to be maintained to avoid the disruption of these MERCS, the Ca^2+^ sensitive motorprotein Miro cease all transportation movements of the mitochondria involved [195]. In the mitochondria PINK1 phosphorylates MFN2, recruits Parkin at the MERCS, allowing Parkin dependent ubiquitination of ER MFN2, promoting the separation of the two organelles and the initiation of mitophagy [196]

Organelle contacts are essential for the maintenance of cellular homeostasis and establish a link that allows inter-organelle signalling and transfer of metabolites [114, 176]. Contact sites refer to areas where two membranes are near each other, but do not merge as the individual organelles maintain their distinct identities. MERCS are dynamic structures that remodel in response to intra and extra cellular signals, affecting the function of both mitochondria and ER [5, 176]. MERCS are relatively stable structures that require the formation of molecular bridges established by interacting proteins anchored in the smooth ER and the mitochondrial outer membrane [5]. MERCS contain a defined subset of proteins involved in tethering membranes, Ca^2+^ homeostasis, lipid transfer, redox balance and mitochondrial homeostasis [5, 40] (Fig. 3). The contacts between ER and mitochondria can be classified as narrow (8–10 nm) and wide (40–50 nm), resulting in different responses against stress and metabolic changes [121].

### Tethering of MERCS

The tethering complexes are essential, structural and reversible bonds that stabilise MERCS [177]. The most recognised MERCS tethering complexes occur between ER mitofusin-2 (MFN2) and mitochondrial MFN2 or ER MFN2 and mitochondrial mitofusin-1 (MFN1) [178]. The MFN tethering complex is dependent on the interaction of MFN2 and PERK on the ER membrane, suggesting a potential role of PERK (and ultimately the UPR^ER^) as a key mediator of MERCS assembly [90]. The interaction of PERK with MFN2 is essential for the establishment of contact sites, inhibition of these components lead to a reduction in the number of MERCS [90, 179] (Fig. 3a). Ablation of MFN2 leads to an abnormal upregulation of the PERK-ATF4-CHOP pathway, resulting in an increase in ROS, abnormal mitochondrial Ca^2+^ transients and altered mitochondrial morphology [179]. Knockdown of PERK in this condition can restore these alterations, demonstrating that PERK is a key regulator of the mitochondrial antioxidant response [179]. Other members of the complexes reported as regulating the tethering of MERCS include the ER vesicle‐associated membrane protein B (VAPB) and the OMM tyrosine phosphatase‐interacting protein‐51 (PTPIP51) [180]. Disruption of these components lead to a delay in Ca^2+^ flux into mitochondria and mitochondrial aggregation [181, 182]. The ER membrane chaperone B-cell receptor-associated protein-31 (BAP-31) can also form a physical and regulatory tether with different mitochondrial proteins [177], such as the mitochondrial fission protein-1 (FIS1), which contributes to the physical tethering and can promote the transmission of apoptotic signals from the ER to mitochondria [183]. Similarly, the interaction of BAP-31 with TOMM40 establish a physical tether that allows BAP-31 to control the transmission of apoptotic signals and regulate mitochondrial homeostasis [184].

### Calcium flux between the ER and the mitochondria

**Fig. 4:**
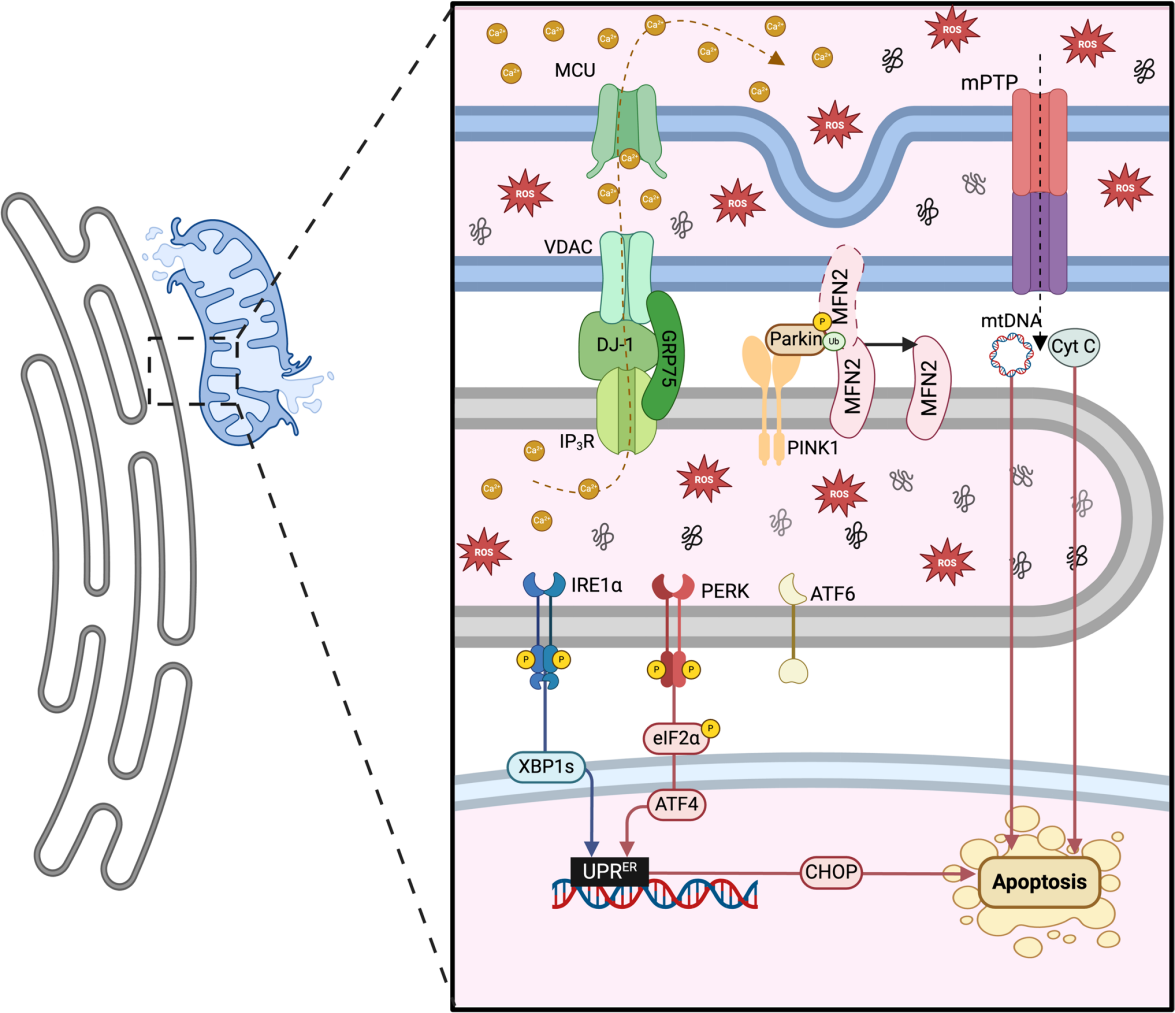
MERCS regulation of cellular signalling in ageing and disease. Disruption of MERCS assembly and disassembly plays a key role in pathophysiological conditions particularly in ageing and age-related diseases. Disrupted Ca^2+^ flow from the ER to mitochondria can result in mitochondrial dysfunction with loss of mitochondrial membrane potential and mitochondrial ROS generation, that result in activation of apoptotic pathways or senescence [40]. Excess Ca^2+^ transfer into mitochondria via IP3R can induce the opening of the mPTP, release of cytochrome c and activation of the caspase signalling cascade and pro-apoptotic pathways [198]. On mitochondria PINK1 phosphorylates MFN2, recruits Parkin at the MERCS, allowing Parkin dependent ubiquitination of ER MFN2, promoting the separation of the two organelles and the initiation of mitophagy [196]. Release of mtDNA through channels such as VDAC (located in or close to MERCS) has emerged as a potential regulator for the inflammatory response [201]

An important function of MERCS is regulation of Ca^2+^ flux between the ER and the mitochondria by the complex that forms between IP3R from the ER and VDAC from the OMM [5, 177]. Ca^2+^ passes through the MCU to reach the mitochondrial matrix [185, 186]. DJ-1 [187] and GRP75 [188] regulate the connection between IP3R and VDAC stabilising MERCS integrity allowing entry of Ca^2+^ into mitochondria [189] (Fig. 3b). It has been recently demonstrated that IRE1a is also involved in regulating ER-mitochondria Ca^2+^ transfer by interacting with IP3R, stimulating mitochondrial respiration and ATP production to maintain energy homeostasis [197]. Ca^2+^ entry into the mitochondrial matrix provides Ca^2+^ to mitochondrial membrane proteins, however in cases of chronic stress it promotes swelling of the mitochondria and the opening of the mPTP that can initiate apoptosis [5, 41]. Some components of the TCA cycle (isocitrate dehydrogenase, oxoglutarate dehydrogenase and pyruvate dehydrogenase) require the binding of Ca^2+^ for their function. The ER poses a much higher concentration of Ca^2+^ (100–500 μM) compared to the cytosol (~ 100 nM), the interaction of mitochondria and ER via MERCS can supply enough Ca^2+^ to mitochondria for stimulating the TCA cycle, resulting in an increase in ATP production [190] (Fig. 3). Excess Ca^2+^ transfer into mitochondria via IP3R can induce the opening of the mPTP, release of Cytochrome c and activation of the caspase signalling cascade and pro-apoptotic pathways [198] (Fig. 4).

### Regulation of mitochondrial homeostasis

**Fig. 5:**
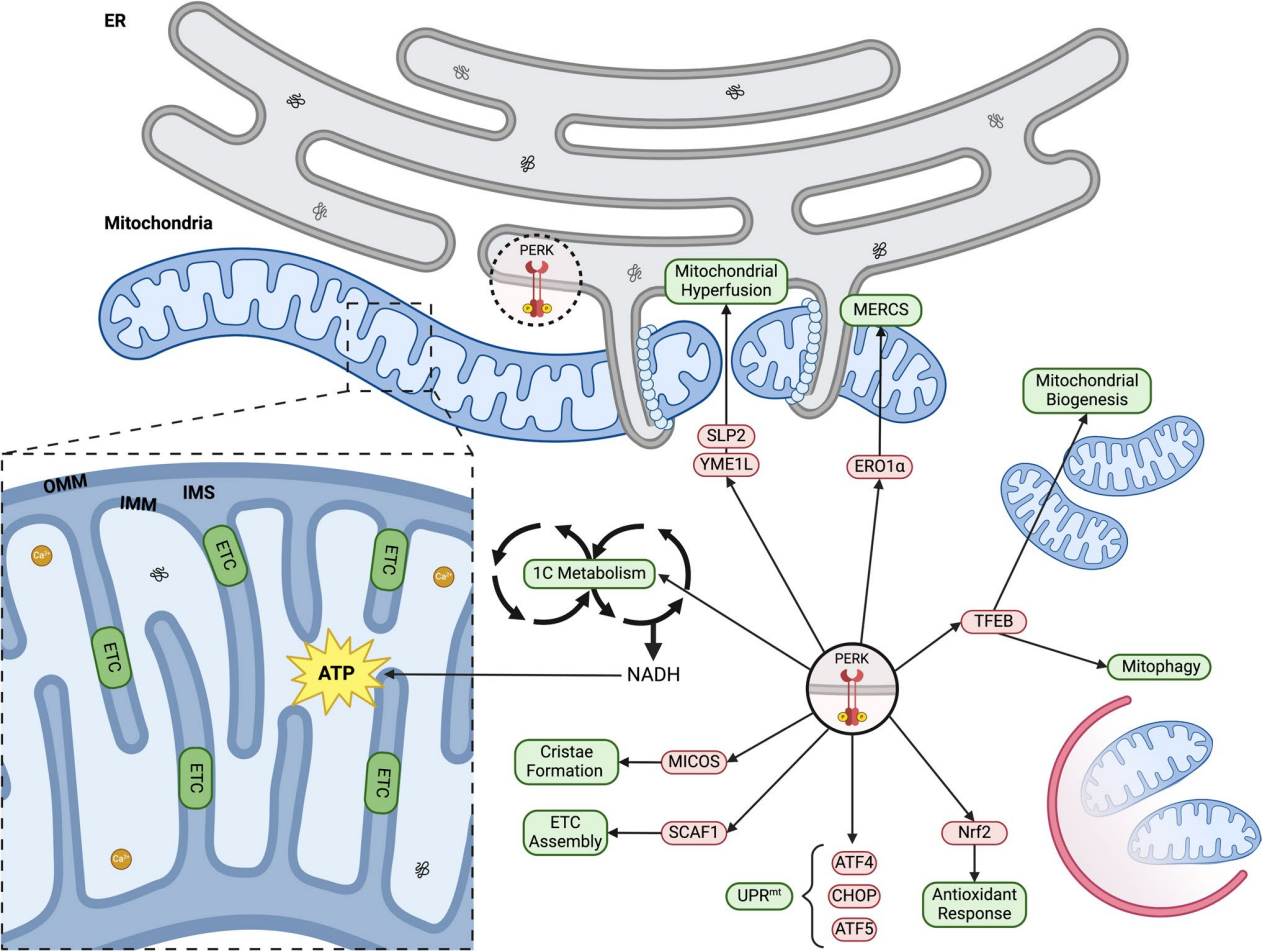
PERK regulation of mitochondrial capacity. PERK is a key regulator of both the UPR^ER^ and the UPR^mt^, that localises at MERCS [90]. The adaptive ER stress response promotes mitochondrial elongation and network establishment [172]. The modulation of mitochondrial metabolism by PERK results in improved cristae formation, assembly of the ETC and oxidative phosphorylation efficiency [220]. **1** PERK regulates the expression of the mitochondrial contact site and cristae-organizing system (MICOS) [221]. **2** The activation of ATF4 by PERK promotes the expression of SCAF1 helps mediate assembly of the ETC [218, 222]. **3** The adaptive UPR^ER^ also promotes one-carbon metabolism [223]. **4** PERK can promote cell survival by increasing antioxidant capacity through the activation of Nrf2 [224]. **5** During the adaptive UPR^ER^ response, there is an upregulation of TFEB [225], which can induce the ISR via activation of ATF4 and CHOP, activate mitophagy machinery and boost mitochondrial biogenesis by promoting expression of PGC1α, TFAM and NRF1 [219]. **6** The formation of PERK-ERO1⍺ complex can restore mitochondrial homeostasis and promote the formation of MERCS [188, 226]. **7** PERK is essential for the activation of UPR^mt^ transcription factor ATF5 [139] and can reduce mitochondrial protein import by promoting the degradation of mitochondrial translocase TIM17A by phosphorylation of eIF2α [227]

Mitochondrial fusion, fission and mitophagy and the organisation of the mitochondrial network regulate mitochondrial function and fuel utilisation [199]. The ER can coordinate these processes by establishing contact sites between ER tubules and mitochondria [111, 191]. The ER inverted formin-2 (INF2) interacts with the OMM actin nucleator Spire1c to polymerise actin filaments and establish close contacts between the two organelles [192]. Actin polymerisation around mitochondria stimulates ER tubules to release Ca^2+^ ions into mitochondria through the VDAC1 channel, triggering the inner mitochondrial membrane to divide [192, 193]. The inner membrane scission is followed by constriction of the outer membrane, which occurs when the cytosolic GTPase DRP1 assemble around mitochondria at the fission site, guided by the OMM receptors FIS1 and MFF [193, 200]. This DRP1 ring constricts with the aid of actin–myosin filaments, resulting in the formation of two daughter mitochondria [191, 193] (Fig. 3). During mitochondrial fission, the original mitochondrion needs to transfer a copy of mtDNA to daughter mitochondria, MERCS mediate the replication and distribution of the mtDNA along the mitochondrial network, in a process that depends on DRP1 [201, 202]. Disruption of mitochondrial dynamics and subsequently mtDNA replication, may result in the release of mtDNA into the cytoplasm and in the generation of an inflammatory response [203, 204]. Considering that the release of mtDNA is thought to occur through channels such VDAC (located in or close to MERCS), and as MFN2 mediates the tethering of ER with mitochondria, contact sites between these two organelles emerge as a potential regulator of the inflammatory response [201] (Fig. 4). If mitophagy is activated, the pre-autophagosome markers ATG14L and ATG5 [205] and the mitophagy regulator PINK1 and Parkin localise to MERCS [206]. In the mitochondria PINK1 phosphorylates MFN2, recruits Parkin at MERCS, allowing Parkin dependent ubiquitination of ER MFN2, promoting the separation of the two organelles and the initiation of mitophagy [196] (Figs. 3 and 5).

It has been proposed that ER tubules guide the position and timing of mitochondria fusion through the tethering with mitochondria [191, 194]. Fusion of the OMM is mediated by MFN1 and MFN2 homodimers [207, 208], while the IMM fusion is regulated by OPA1 [209]. During mitochondrial fusion the contact sites between the tubules and the mitochondria need to be maintained to avoid the disruption of MERCS and decrease mitochondrial motility [210]. In yeast during mitochondrial fusion, the Ca^2+^ sensitive motorprotein Miro, is involved in both actin filament and microtubule transport, that ceases all transportation movements of the mitochondria involved [195] (Fig. 3).

### Redox Regulation of MERCS

The connection established by MERCS between the ER and mitochondria implies that disruption of redox homeostasis in one organelle will affect the other, generating a regulatory hub. It has been reported that ROS production in mitochondria leads to an exacerbation of ER stress, suggesting the existence of a feed-back loop that generates ROS in both organelles [211]. Within MERCS there is a constant production of ROS, generated from the oxidative protein folding activity of the ER chaperone ERO1α and the ER NADPH oxidase activity of NOX4 [212]. The presence of ROS within MERCS generates redox nanodomains between the two organelles, in a Ca^2+^-dependent process, allowing for effective redox crosstalk [213]. Targeting a H_2_O_2_-specific fluorescent probe to MAMs, it was reported that these redox nanodomains promoted IP3R-mediated Ca^2+^ release via MERCS,resulting in the swelling of the mitochondrial matrix, reduction of the cristae and release of H_2_O_2_ [213].

PERK is a key regulator of both the UPR^ER^ and the UPR^mt^ and localises at MERCS [90, 214]. Mouse embryonic fibroblasts with PERK knocked out, have a disrupted MERCS network, altered ER morphology, disrupted redox signalling and impaired Ca^2+^ transport [215, 216]. PERK is a regulatory signalling hub that monitors stress in both organelles and its Cys216 can be reversibly oxidised allowing formation of covalent interactions with ERO1α and tightening of MERCS [90, 217]. UPR^ER^ and UPR^mt^ establish a crosstalk in response to proteotoxic stress through PERK activation, regulating the coactivation of CHOP and ATF4 and increasing the expression of ATF5, promoting the translation of ER and mitochondrial chaperones to alleviate proteotoxic stress [217]. As mentioned, the UPR can be an adaptive or maladaptive response depending on stress intensity and duration, that can impact mitochondrial morphology and function [172, 218, 219].

The UPR^ER^ effects on mitochondrial morphology go through different stages: early ER stress (30 min) induces mitochondrial fragmentation, MERCS formation and Ca^2+^ influx into mitochondria; adaptive ER stress (6 h) promotes mitochondrial elongation and network establishment, improving oxidative phosphorylation efficiency [228], known as stress-induced mitochondrial hyperfusion [172] (Fig. 5). Maladaptive ER stress (24 h or more) triggers apoptosis through mitochondrial fragmentation and opening of the mPTP [172, 220, 229]. Inhibition of PERK or p-eIF2α during the adaptive UPR^ER^ stage induced the blockage of mitochondrial hyperfusion and fragmentation of the mitochondrial network [172], indicating that communication between the ER and mitochondria is mediated by the PERK-eIF2α axis.

Adaptive UPR^ER^ protects the cells against oxidative damage though the activation of PERK, which can boost the production of ATP [218] and activation of the antioxidant response [223]. The modulation of mitochondrial metabolism by PERK results in improved cristae formation, assembly of the ETC and oxidative phosphorylation efficiency [220]. During adaptive UPR, PERK phosphorylates N-acetyl-glucosamine transferase OGT, which can activate TOM70 stimulating the import and assembly of the mitochondrial contact site and cristae-organizing system (MICOS) [221] (Fig. 5). The activation of ATF4 by PERK promotes the expression of SCAF1, a protein that mediates the assembly of the ETC [218, 222] (Fig. 5). It has been reported that cells with a missense mutation in complex I NADH ubiquinone oxidoreductase, were able to recover the assembly of the super complexes by pharmacologically activating PERK [218]. As a counter measure to stress, the adaptive UPR^ER^ promotes one-carbon metabolism, in a process mediated by PERK [223]. One-carbon metabolism links the methionine and folate pathways through the interconversion of Serine and Glycine providing one carbon units for biosynthesis and reducing power in the form of NADH and NADPH [230] (Fig. 5).

PERK can promote cell survival by increasing antioxidant capacity through the activation of nuclear factor erythroid 2-related factor 2 (Nrf2) [224] (Fig. 5). PERK phosphorylation of Nrf2, releases it from Keap1 and subsequent translocation to the nucleus, initiating the transcription of numerous antioxidant genes, including thioredoxins, glutathione synthetase, glutathione S-transferase, and ferritin [231]. PERK silencing resulted in disrupted Nrf2 activation, an increase in ROS and an impairment of mitochondrial bioenergetics [232]. Key interactions of PERK that help determine mitochondrial capacity are established with TFEB, ERO1⍺ and the UPR^mt^ [90, 139, 219]. During the adaptive UPR^ER^ response, there is an upregulation and nuclear translocation of TFEB [225]. TFEB can activate the ISR via ATF4 and CHOP, promotes the activation of mitophagy machinery and boost mitochondrial biogenesis by the expression of PGC1α, TFAM and NRF1 [219] (Fig. 5). The formation of a PERK-ERO1⍺ complex can restore mitochondrial homeostasis and promote the formation of MERCS by increasing tethering via GRP75 and MFN2 [188, 226] and stimulating Ca^2+^ transfer to increase mitochondrial capacity [90] (Fig. 5). PERK is essential for ATF5 activation and UPR^mt^ [139], and can reduce mitochondrial protein import by promoting the degradation of mitochondrial translocase TIM17A by phosphorylation of eIF2α [227] (Fig. 5).

### MERCS in ageing and disease

The dynamic nature of MERCS in terms of assembly and disassembly are determined by intracellular cues, allowing adaptation to the intracellular environment for both cell survival associated with increased metabolism but also potentially triggering the collapse of mitochondrial membrane potential resulting in apoptosis or senescence. MERCS can regulate Ca^2+^ homeostasis, redox signalling and lipid transfer, providing signalling hubs that can modulate mitochondrial dynamics, apoptosis, protein homeostasis and inflammation [40]. As a result, disruption of MERCS assembly and disassembly is thought to play a key role in pathophysiological conditions particularly in cancers and age-related diseases. In proliferating cells with high anabolic demand, mitochondrial fission predominates over mitochondrial fusion, MERCS can help determine mitochondrial morphology and allow efficient transfer of Ca^2+^ and other metabolites to mitochondria during proliferation. The accumulation of cells that have entered cell cycle arrest or senescence in ageing tissues is well documented [233]. MERCS assembly and disassembly provide a regulatory role in determining cell fate. Disrupted Ca^2+^ flow from the ER to the mitochondria can result in mitochondrial dysfunction with loss of mitochondrial membrane potential and increased mitochondrial ROS generation, resulting in activation of apoptotic pathways or senescence [40]. Senescent cells accumulate during ageing, an increase in the cell capacity to remove senescent cells results in delayed aging and improves both lifespan and health-span [234]. It has been reported that the exposure to pro-senescent stressors or other stimuli can alter the number of MERCS [40, 235]. An aberrant increase in MERCS, during ageing, can result in the accumulation of Ca^2+^ in the mitochondria, activation of the p53/p21 and p16/Rb pathways, leading to cell cycle arrest and Senescence-Associated Secretory Phenotype (SASP) partially driven by NF-κB [40, 235]. Senescence of endothelial cells is considered to be a risk factor related to the development of cardiovascular disease and can contribute to disrupted vascular tone and angiogenesis [236]. It has been demonstrated in an in vitro model of endothelial cell ageing that increased MERCS formation result in an increase in Ca^2+^ transfer, altering mitochondrial bioenergetics and cell senescence [237]. Most studies would indicate senescence is associated with increased MERCS formation and elevated mitochondrial Ca^2+^, however decreased MERCS formation could also be a pro-senescent signal [40]. However, it is clear that not only the abundance of MERCS is important but also the width of the interface between the ER and OMM, where loose junctions (~ 25–40 nm) promote Ca^2+^ transfer and tight junctions (~ 10 nm) inhibit Ca^2+^ transfer between the organelles [121].

Changes in MERCS formation is context dependent and distinct between cell types, with a number of pathologies reporting increased MERCS formation and others decreased MERCS formation. In cancerous cells, increased Ca^2+^ uptake in the mitochondria can promote metabolism and tumorigenesis, however excessive Ca^2+^ uptake can induce cell death [238]. In neurodegenerative diseases such as Alzheimer disease and Parkinson disease, increased MERCS have been reported [239]. Mitochondrial dysfunction in neurodegenerative diseases, are associated with the loss of neuron structure and function and altered protein composition of MAMs, required for the scaffolding of MERCS and ultimately disrupted mitochondrial turnover [240–242].

### Skeletal muscle and adaptive UPR signalling

In almost all eukaryotic cells the ER is an essential organelle for protein synthesis and folding, lipid and sterol synthesis, as well as a depot for the storage of Ca^2+^. The contraction and relaxation of skeletal muscle depends on the on the release and uptake of Ca^2+^ from the sarcoplasmic reticulum (SR). The SR has been described as a fully differentiated domain of the muscle ER and it is recognised that the ER and SR are a continuous membrane system of different specialised regions [243, 244]. The SR contains a number of recognised ER proteins, although at a relatively lower concentration and it was proposed that during myogenic differentiation there is ER expansion that is engulfed by myofibrils [243, 245].

### UPR^ER^ activation during myoblast differentiation

UPR^ER^ activation is crucial for muscle stem cell homeostasis, myogenic differentiation, exercise adaptation and skeletal muscle regeneration after injury [246]. Myogenesis is a complex and tightly regulated process that involves the selection of multipotent mesodermal cells to produce myoblasts, their exit from the cell cycle and differentiation into myotubes [247]. During muscle differentiation a population of myoblasts, that are differentiation-incompetent or less resistant to stress, will undergo selective apoptosis [248]. This process is thought to be mediated by the UPR^ER^ and it is crucial for skeletal muscle development [247]. The UPR^ER^ plays an essential role in this process by controlling the induction of caspase-12, promoting a caspase signalling cascade that results in selective apoptosis [249]. Markers of the UPR, such as ATF6, CHOP, and BiP, are upregulated during myogenesis and it has also been demonstrated that pharmacological induction of ER stress increased apoptosis in myoblasts, leading to improved myogenesis [249, 250]. Pharmacological induction of ER stress (using the N-glycosylation inhibitor tunicamycin and the SERCA inhibitor thapsigargin) in myoblasts lead to an increase in cell apoptosis, however the remaining myoblasts differentiated more efficiently into myotubes [250].

### Redox and adaptive UPR^ER^ in skeletal muscle

Exercise is one of the most effective and beneficial interventions for overall health. Exercise can improve insulin sensitivity, cardiovascular health and help maintain muscle mass and function [251]. Regular exercise has been shown to reduce oxidative stress, inflammation, and reverse mitochondrial and ER dysfunction [252]. The changes in Ca^2+^ flux during muscle contractions has been associated with the formation of contact sies between the sarcoplasmic reticulum and mitochondria [244]. During contractile activity there is localised endogenous ROS generation that is required for the activation of specific signalling pathways required for the adaptive response to exercise [253, 254]. Temporal endogenous ROS generation is also necessary for the repair and activation of quiescent satellite cells following muscle injury [255]. Fluctuations in Ca^2+^ homeostasis, together with an altered redox environment are linked to the activation of all 3 branches of the UPR^ER^ following exercise with downstream signalling effects on mitochondrial dynamics [113, 256].

Although chronic ER stress can activate cell death pathways, recent research suggests that low levels of ER stress may potentially benefit cells by inducing an adaptive UPR that can reduce the harmful consequences of accumulating misfolded proteins [231]. Physical exercise generates a physiological stress and activation of UPR^ER^ pathways, several studies have demonstrated that acute exercise is characterised by an increase in BiP translation and eIF2 phosphorylation [257–259]. As a result, regular exercise can inhibit the activation of pro-apoptotic pathways, maintaining or decreasing the levels of BiP, PERK, IRE1a and CHOP including downstream UPR^ER^ components such as ATF4 and XBP1 [260, 261]. Mitochondria are also affected by contractile activity in skeletal muscle, it has been demonstrated that exercise plays a key role in mitochondrial adaptation to stress, promoting mitochondrial biogenesis and mitophagy [254]. PGC-1α is activated in skeletal muscle in response to exercise, promoting mitochondrial biogenesis and the adaptative response to exercise [256]. It has also been reported that PGC-1α regulates the expression of ATF5 [262], providing a link between activation of the UPR and mitochondrial biogenesis.

In skeletal muscle there are distinct populations of mitochondria, subsarcolemmal and intermyofibrillar, providing the ATP required for sustaining contractions and membrane potential. Mitochondria are in close contact with the SR and it has been proposed that MERCS are essential for maintaining muscle homeostasis [263]. MERCS impairment in skeletal muscle is associated with ageing and muscle wasting, caused by the downregulation of SR-mitochondria Ca^2+^ transport proteins IP_3_R, VDAC, and GRP75 [264]. Disruption of Ca^2+^ transits between the SR and the mitochondria may contribute to the decline in muscle performance during ageing [264–266]. In single adult skeletal muscle fibres, pharmacologically opening of the mPTP resulted in increased mtROS and caspase activation, leading to muscle fibre atrophy [267]. In striated muscle, the partitioning of ER/SR and mitochondria is highly organised and as a result MERCS formation are considered more ordered compared to proliferating cells [264]. Disrupted Ca^2+^ homeostasis is thought to play a role in the age-related loss of skeletal muscle function and muscular pathologies. Decreased MERCS formation has been reported with age [268] and depletion of MERCS are associated with muscular dystrophy [263]. In pathophysiological conditions, disrupted inter-organelle communication between mitochondria and ER results in altered contact sites, potentially resulting in a resistance to mitochondrial degradation, accumulation of dysfunctional mitochondria, release of proinflammatory mtDNA and an amplification of the pathophysiological response. Energetic stress and subsequent AMPK activation has been demonstrated in cell models to promote autophagy and MERCS formation [269]. From a physiological perspective introducing an exercise protocol that involves extensive cytoskeletal remodelling and energetic stress, that can promote UPR activation and induce mitochondrial remodelling, would ultimately result in an improved bioenergetic profile. This beneficial adaptive response may be facilitated by increased formation of MERCS.

## Conclusions

The intricate crosstalk between the ER and mitochondria can be mediated by MERCS, providing an effective conduit for cell signalling and facilitating the exchange of information and metabolites. There are still a large number of outstanding questions in the field in relation to how the activation of the UPR following ER stress mediates the assembly and disassembly of MERCS. Similarly, it is still uncertain how MERCS influence the UPR and how alterations in MERCS may impact the cell's ability to respond to ER stress. It is clear from studies using a variety of tissues that MERCS directly impact and determine mitochondrial function and dynamics. As a result, MERCS are critical regulators of cell fate under conditions of stress, determining whether the cell will undergo an adaptive response, proliferate, initiate apoptosis or undergo cell cycle arrest and senescence. Disruption of MERCS formation could result in ER stress response dysfunction, where there is impaired UPR activation and failure to activate the appropriate arms of the UPR and subsequent downstream signalling effects. Modulation of MERCS formation could potentially be a valuable therapeutic approach in order to exacerbate mitochondrial Ca^2+^, increased ROS formation to potentially sensitise senescent cells to apoptosis [270].

## Data Availability

Not applicable.
